# Assessment and diffusion of medical innovations in France: an overview

**DOI:** 10.1080/20016689.2018.1458575

**Published:** 2018-04-04

**Authors:** Amélie Dubromel, Loïc Geffroy, Gilles Aulagner, Claude Dussart

**Affiliations:** a Pharmacy Department, Hôpital d’Instruction des Armées Desgenettes, Lyon, France; b Laboratoire Parcours Santé Systémique, Université Claude Bernard Lyon, Lyon, France; c Pharmacy Department, Hospices civils de Lyon, Groupement hospitalier Est, Bron, France

**Keywords:** Innovation, health care, decision making, health technology assessment, pharmacoeconomics, market access

## Abstract

**Background:** In France, a significant part of health expenditure is publicly funding. This put a heavy burden on society. In an economic context requiring tight control of public spending, it seems relevant to control the diffusion of medical innovations. That is why health technology assessment is subject to an increasing interest at national level for management and approval decisions. This article provides an overview of the assessment and diffusion of medical innovation in France. **Method:** The data are extracted from French authorities or organisations websites and documents and from French legislative texts. In addition, regarding discussion, a search in MEDLINE database was carried out. **Results:** An overview of the assessment and diffusion of medical innovation in France is given by presenting the different types of medical innovations according to French health system definition (I); introducing French authorities participating to health technology assessment and describe assessment procedures of medical innovations (II); and giving details about market access process of innovative health product in France (III). Key opportunities and challenges of medical innovation assessment and diffusion in France are discussed at the end of this article. **Conclusion:** In France, medical innovation is considered as a crucial component for quality of care and performance of healthcare system. The aim of health technology assessment is to promote a secure and timely access to innovation for patients. Nevertheless, it appears necessary to improve regulatory mechanisms.

## Background

In France, a significant part of health expenditure is publicly funding. This put a heavy burden on society. As a matter of fact, in the French economy tables, published in 2017 by the French National Institute for Statistics and Economic Studies (INSEE), it is reported that the current health expenditure (*Dépense courante de santé* – *DCS*) of 2015 accounted for 12.0% of gross domestic product (GDP) [1]. In an economic context requiring tight control of public spending, and particularly health spending, it seems relevant to manage the diffusion of expensive medical innovations either because of their individual cost or because of their volume of use [2]. It reinforces the significance of health technology assessment at national level, with the aim of controlling medical innovation diffusion. This article provides an overview of the assessment and diffusion of medical innovation in France by presenting the different types of medical innovations according to French health system definition (I); introducing French authorities participating to health technology assessment and describe assessment procedures of medical innovations (II); and giving details about market access process of innovative health product in France (III). Key opportunities and challenges of medical innovation assessment and diffusion in France are discussed at the end of this article.

## Method

The data presented in this article are extracted from French authorities or organisations websites and documents and from French legislative texts. As far as possible, the most recent and detailed data were selected. The starting point for the research related to definition, assessment and market access processes of medical innovation in France was the French Ministry of Health and Solidarity website. In addition, regarding challenges and opportunities section, a search in MEDLINE database (*Pubmed, United States National Library of Medicine*) was carried out over a period ranging from 01/01/1990 to 03/05/2017. The following key words were used: ‘*decision support tool*’, “*decision support model*", “*decision support technique*", “decision-making", “*health technology assessment*", “*HTA*", “criteria" and “systematic review".

## Definition of medical innovation in France

According to the 3rd version of the Oslo manual, established by the Organisation for Economic Co-operation and Development (OECD), ‘an innovation is the implementation of a new or a significantly improved product (good or service), or process, a new marketing method or a new organisational method in business practices, workplace organisation or external relations’ [3]. In the section on Medico-Economic Research Program (*Programme de recherche médico-économique – PRME*) from instruction no. DGOS/PF4/2014/33 of 28 January 2014, innovation is defined as ‘diagnostic, therapeutic or screening health technology, which is in first diffusion, marketing or commercialization phase, and of which efficiency and safety have been validated in clinical research. For health product, it is specified that innovative health technology need to have a marketing authorization or a CE marking’ [4]. The definition provided by the International Network of Agencies for Health Technology Assessment (INAHTA) has been selected for specification of health products, i.e., all test, device, medicine, vaccine, procedure, program or system developed to prevent, diagnose, or treat medical conditions; promote health; provide rehabilitation; or organize healthcare delivery [5].

The definition of innovation includes concepts of novelty and change. It is therefore relevant to consider the relationship between nature of innovation and scope of change. It is then crucial to distinguish disruptive innovations from incremental innovations. The concept had been explained by Clayton M. Christensen in his book entitled *The innovator’s dilemma* [6], published in 1997; he describes disruptive innovation as the one which creates, transforms or destroys a market. It involves discovery of new consumers or consumption pattern, and development of new disruptive technology or corporate governance models. In contrast, incremental innovation result from the emergence of a new superior performance technology, but it is not associated with transformation or creation of a market. A report of Economic, Social and Environmental Council (*Conseil économique, social et environnemental – CESE*) takes up this concept. In that report, disruptive innovation are defined as those that introduce a significant change, can cure or bring a profit of residual life expectancy for example. Incremental innovations, for their part, improve the treatment of a disease without involving shift in process of care [7]. In the health field, disruptive innovations are most likely to generate a significant variation in expenditures, particularly through change in medical strategies, care pathways, or even health pathways which include care, prevention, social and medico-welfare accompaniment and home return or care assistance [8].

The French Ministry of Health and Solidarity considers medical innovation at large, whether technical (medical device, drug, procedure) or organisational, as an essential component in the quality of care and the performance of healthcare provision [2]. Despite rich literature on the subject, there is no consensus on the specification of medical innovations [9]. The following aspects have been selected.

### Innovative medical equipment and devices

The French National Agency for Medicine and Health Products Safety (ANSM) offers to note the degree of novelty of medical devices depending on their technical and clinical innovation degree [10]. Distinction between concepts of disruptive innovation and incremental innovation is included. Indeed, devices that ‘affect existing technologies in the health field, and that may definitely replace them’ are defined in opposition to devices that ‘show technological improvement in comparison with other devices’. We cite for example the use of MRI scanner for the diagnostic of stroke [11].

### Innovative drugs

The French National Authority for Health (HAS) consider that a new drug is innovative if one or more of the conditions mentioned bellow are fulfilled:

- Drug associated with new type of care
- Drug that may bring a clinically significant advance compared to the means available
- Drug that meet a need that is not or not sufficiently covered [12].

When authorized and marketed, drugs enabling brain clot thrombolysis were considered as innovative [11].

### Innovative surgical and medical procedures

Medical or surgical procedure is innovative if it meets the four requirements, set out in instruction no. DGOS/PF4/2015/258 of 31 July 2015 [13]. Therefore, it shall:

- Display a novelty feature, other than a single technical evolution;
- Be in an early diffusion phase;
- Be examined with a study of risks associated with its use for patients and healthcare professionals;
- Show significant clinical benefit allowing to meet medical need, which is insufficiently or not covered;
- Or show clinical benefit allowing reducing significantly health expenditures.

The use of thrombectomy, which is an endovascular surgery that aim to mechanically remove the blood clot is a good example of innovative surgical procedure [11].

### Innovative organisational systems

Organisational innovation is defined in the section on the French Performance of the Healthcare System Research Program (*Programme de recherche sur la performance du système de soins* – *PREPS*) from instruction no. DGOS/PF4/2014/33 of 28 January 2014 [4], as the research on new organisation of care provision, with the aim of:

- Developing new types of patient management,
- Improving performance of healthcare providers,
- Improving quality of practices and care,
- Optimizing care pathways.

The emergence of innovative organisational systems is closely associated with the onset of technical medical innovation. The care of Cerebral Vascular Accident is a good example. The creation of neurovascular units (NVUs) was closely tied up with the diffusion of innovative drugs, medical imaging equipment and surgical procedure previously cited [11].

## Authorities and medical innovation assessment in France

### French authorities and health technology assessment (HTA)

The French National Authority for Health (HAS) is responsible of the evaluation of medical innovations. The three following committees carry out this mission:

- The Transparency Committee (*Commission de transparence* – *CT*) [14];
- The Medical Device and Health Technology Evaluation Committee (*Commission Nationale d’Évaluation des Dispositifs Médicaux, des Actes et des Technologies de Santé – CNEDiMTS*) [15];
- The Economic and Public Health Evaluation Committee (*Commission d’Evaluation Economique des Produits de Santé – CEESP*) [16].

Furthermore, innovation and clinical research office from the Directorate of Health Care Supply (*Direction Générale de l’Offre de Soins* – *DGOS*) promotes every year research programs through the publication of several calls for tender, and contributes to data acquisition. Those data are essential to health technology assessment conducted by the committees mentioned above [17].

### Assessment of medical innovations in France

#### Ex-ante evaluation

Assessment of medical innovations starts with clinical feasibility studies, which explore benefit/risk ratio, such as phase I and II non-comparative studies [2]. Then, safety and efficiency must be evaluated [2]. The studies carried out are comparative and randomized as far as possible. Such studies are eligible for Hospital Clinical Research Program (*Programme hospitalier de recherche clinique* – *PHRC*), which is financed by Teaching, Research, Reference and Innovation Missions (*Mission d’enseignement, de recherche, de référence et d’innovation – MERRI*) credits [2,4].

Assessment of medical innovations continues with clinical utility, efficiency and medico-economic studies [2]. Those types of studies are eligible for Medico-Economic Research Program (PRME), which is financed by MERRI credits [2,4].

Simultaneously, assessment of organisational medical innovations may be done and funded through the Performance of Healthcare System Research Program (PREPS) [2,4]. Indeed, the introduction and then the use of innovative health technologies are advantageous for patient care, particularly through the improvement of care pathways. That is why researches that contribute to optimize health care performance and to better understand impact of organisational changes, work practices, health policies and regulation tools, are encouraged in parallel to health technologies assessment [4,18].

**Figure 1.**
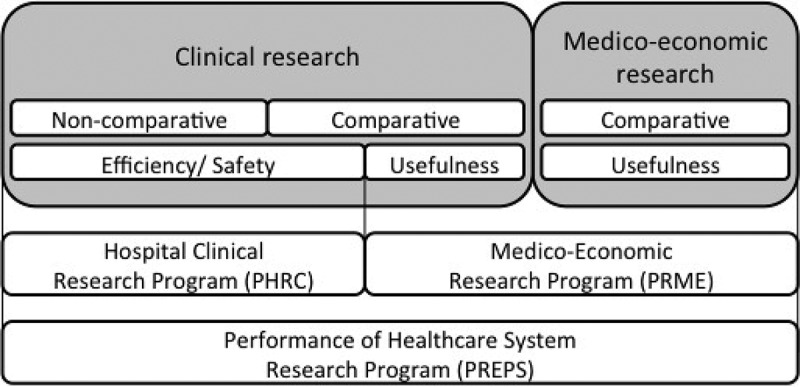
Ex ante evaluation of medical innovations in France.

Figure 1 shows ex ante evaluation process of medical innovations in France [2].

#### Evaluation carried out by the French National Authority for Health (HAS)

Some of the most primary missions of the HAS, as national Health Technology Assessment agency, are to periodically assess expected clinical benefit of health products, acts or health services compare with their provided clinical benefit and support decision making related to registration, reimbursement and management by the French National Health Insurance (*Assurance maladie*) of products, acts and health services as well as related to specific conditions of care for individuals with long-term condition [19].

Therefore, The Transparency Committee (TC) evaluates the Actual Clinical Benefit (ACB) (*Service médical rendu* – *SMR*) and the Clinical Added Value (CAV) (*Amélioration du service medical rendu* – *ASMR*) of drugs [20]. The CNEDiMTS assesses ACB (*Service attendu* – *SA*) and CAV (*Amélioration du service attendu – ASA*) of certain medical devices [21].

There are 3 levels of ACB (substantial, moderate and low). The level is determined based on severity of disease or condition, efficacy, adverse events, intended role in therapeutic strategy in comparison with other available therapies and public health benefit (*Intérêt de santé public* – *ISP*). ISP notion considers public health needs, impact on population health, impact on healthcare system organisation and impact on public health programs and policies [21,22]. There are 5 levels of CAV (major, substantial, moderate, minor and no improvement). The level is attributes based on comparative efficacy and safety data with regards to available therapies.

In addition, an efficiency opinion is provided by the Economic and Public Health Evaluation Committee (*Commission évaluation économique et santé publique* – *CESSP*), when an economic lightening is needed. This assessment should be carried out if major or substantial or moderate CAV is claimed or is attributed and if a significant impact is expected on the Health National Insurance expenditures. Therefore, this evaluation is exclusively conducted for medical innovations. Efficiency opinion provide by CEESP supplements medical expertise issued by the TC and the CNEDiMTS [16].

#### Ex-post evaluation

It is not unusual that HAS give a favorable opinion for the reimbursement of a medical innovation while it remains uncertainties regarding his use. The doubts may be related to long-term safety or efficiency, effectiveness, respect of indications, or may concern a risk of misuse or unjustified expenditures. In such case, the HAS can apply for post-registration studies. These studies are mentioned in article L163-18 of the French Social Security Code (*Code de la Sécurité Sociale* – CSS) [23]. They are supported by the company marketing the innovation [2]. They tend to gather complementary data, in real use conditions, about treated populations, treatment duration, compliance, benefits, impact on other therapeutic strategies or organisation of healthcare [22]. Those studies can be associated with restrictive conditions regarding diffusion, in application of section L.1151-1 of the French Public Health Code (*Code de la santé publique –* CSP), that may affect a certain occupational category and/or some technical conditions and/or some health facilities [2,24].

## Market access process of innovative health product in France

### Price-setting

One of the main mission of the French Economic Committee for Health Products (*Comité économique des produits de santé* – *CEPS*) is price-setting for medical innovation. This committee is an interministerial body, which is under the joint authority of health, social security and economy ministers [25]. This financial parameter is determined, after negotiation, on the basis of the opinion provided by the Transparency Committee or the CNEDiMTS and the report of the CEESP if economic lightening was necessary. Price-setting may be subject to an agreement between the company marketing the innovative product and the CEPS [25].

### Reimbursement decision-making process

If the HAS opinion is favorable to innovation reimbursement by the French National Health Insurance, the medical innovation is registered on a list. With regard to drugs, it is a list mentioned in article L.162-17 of the French Social Security Code (*Code de Sécurité Sociale* – *CSS*) [26]. With regard to medical devices, it is a list of products and services qualifying for reimbursement (*Liste des Produits et Prestations Remboursables* – *LPPR*) mentioned in article L.165-1 of the French CSS [27]. Registration decision as well as reimbursement rate are acted by the French National Union of Health Insurer Funds (*Union Nationale des Caisses d’Assurance Maladie* – *UNCAM*) [22]. Reimbursement rate is correlated with ACB level.

Regarding diffusion of health products in hospitals, their cost is in most cases integrated to Hospital Stay Related Groups (*Groupes Homogènes de Séjours* – *GHS*). We note that for drugs, their purchase and use at hospital are conditional upon their registration on a list for access to Community Pharmacy [28]. However, when the cost of an innovative drug or medical device is too high for being integrated to the associated GHS, an individual funding in addition to GHS (so-called *en sus*) is intended. In this case the CEPS may decide to register an innovative drug or medical device on a specific list established in article L.162-22-7 of CSS [29]. Involved medical devices are those registered on title III of the LPP (implantable medical devices) [30].

**Figure 2.**
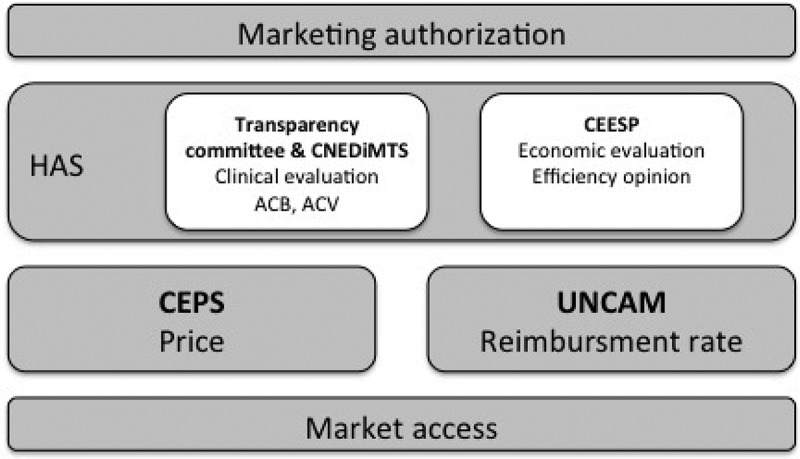
French organisations involved in assessment market-access process of medical innovations.

Figure 2 shows all French organizations involved in assessment and market process of medical innovations.

## Key opportunities and challenges of medical innovation assessment and diffusion in France

### Early and derogatory access opportunities

Medical innovations contribute to improve patient health condition, quality and safety of care, and performance of healthcare delivery [2]. Early and derogatory market access allow for an anticipated provision of innovations for patients, while controlling associated risks as long-term safety, misuse and unjustified expenditures [2].

An early diffusion (anterior to the evaluation conducted by the HAS) is possible according to some conditions for certain innovative drugs. This early diffusion tends to be derogatory and temporary and contains conditions, which include the conduct of clinical and economic trials. This opportunity applies only to drugs and is called Temporary Use Authorization (*Autorisation temporaire d’utilisation* – ATU). Agreement terms are described in article L.5121-12 of the CSP [31]. This early diffusion device is funded with MERRI credits.

With regard to innovative medical devices, if ACB was evaluated as insufficient by the HAS, but that available data show a potential interest of innovation; they can be eligible for innovation pass (*Forfait innovation*), established in article L.165-1-1 of the CSS [32]. It is a controlled diffusion program conditioned to the conduct of complementary studies, particularly clinical studies or eventually economic ones. These studies are supported by the industrial. With some exceptions, any innovation which have been enrolled, before the evluation of the HAS, in a Research Program cannot benefit from innovation pass. During the study and until the decision regarding registration, innovation is funded by the objective of the French National Health Insurance expenditures, which is common to medicine, surgery, obstetrics and odontology activities (*Objectif des dépenses de l’assurance maladie commun aux activités de médecine, chirurgie, obstétrique et odontology* – *ODMCO*) [2]. This diffusion opportunity tends to be derogatory and temporary.

**Figure 3.**
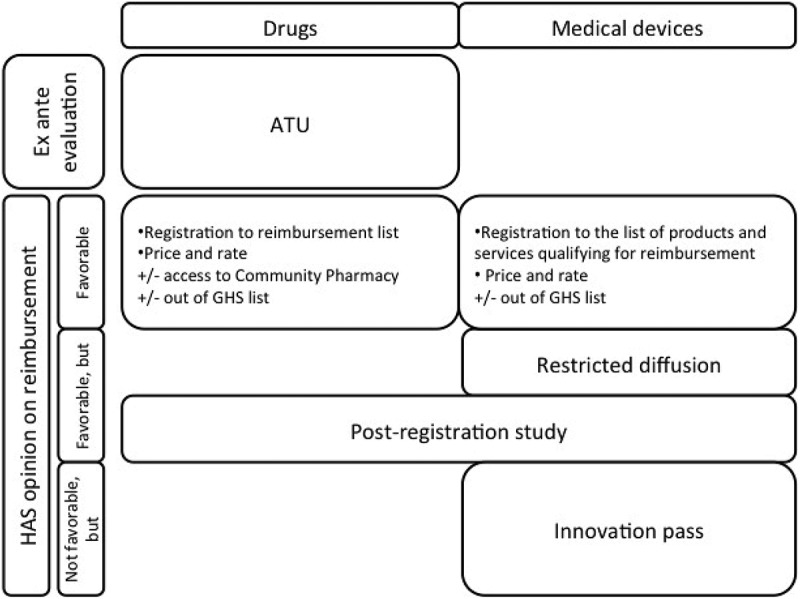
Market-access process and opportunities of early or derogatory diffusion of medical innovations in France.

Figure 3 resumes market access process and opportunities of early and derogatory diffusion of medical innovations in France [2].

### Challenges

From the perspective of national organisations involved in medical innovation assessment and market-access the main challenge is to find balance between access to innovation and affordability. In France, there are many initiatives to support innovation including research programs and early or derogatory diffusion [33]. However, it appears necessary to improve regulatory mechanisms and in particular to develop specific procedures regarding disruptive innovations [7].

Medical innovations are likely to lead to changes in medical strategies, care pathways, or even health pathways. Development of e-health solutions, like telemonitoring for patient with dialysis-stage renal disease or teleexpertise in cancerology, are good examples of innovative organisation that can upset patient care [34,35]. Nevertheless, it is crucial to evaluate impact of changes induced [36]. That is why, the description of organisational impact benefits from an increasing interest [37]. If the organisational dimension still remains currently poorly studied, it is promoted by hospital decision-makers and should being developed in the nearly future [38].

## Conclusion

In France, medical innovation is considered as a crucial component for quality of care and performance of healthcare system. The different research programs allow promoting the emergence of innovative technologies or organisations. Health technology assessment is conducted in order to secure access to innovations for patients. Early and/or derogatory initiatives have been created to accelerate access to innovations for the patient while keeping a high level of security. In fact, these opportunities contain a control of associated risks as long-term safety, misuse and unjustified expenditures. Nevertheless, it appears necessary to improve regulatory mechanisms.

In France, Health Technology Assessment relies on clinical criteria as efficacy and safety and if necessary on economic criteria. However, medical innovations are likely to lead to changes in medical strategies, care pathways, or even health pathways. If the organisational dimension still remains poorly studied, his inclusion in the evaluation process benefits from an increasing interest and should being developed in the nearly future.
